# Potential Impacts of Hydralazine as a Novel Antioxidant on Cardiovascular and Renal Disease—Beyond Vasodilation and Blood Pressure Lowering

**DOI:** 10.3390/antiox11112224

**Published:** 2022-11-11

**Authors:** Ting-Ting Chang, Jaw-Wen Chen

**Affiliations:** 1 Department and Institute of Pharmacology, School of Medicine, National Yang Ming Chiao Tung University, Taipei 112304, Taiwan; 2 Healthcare and Services Center, Taipei Veterans General Hospital, Taipei 112201, Taiwan; 3 Division of Cardiology, Department of Medicine, Taipei Veterans General Hospital, Taipei 112201, Taiwan; 4 Cardiovascular Research Center, National Yang Ming Chiao Tung University, Taipei 112304, Taiwan

**Keywords:** cardiovascular disease, kidney disease, anti-inflammation, antioxidation, hydralazine

## Abstract

Hydralazine is a traditional antihypertensive drug that was developed several decades ago. Its most well-known effect is blood pressure lowering by arterial vasodilation. While mainly used an adjunct treatment for clinical hypertension or chronic heart failure, this old drug has also shown potential as a repurposing drug for the atherosclerosis vascular disease and various kidney diseases. Recent experimental studies suggest that hydralazine exerts antioxidative, anti-apoptotic, and HIF-1α stabilization effects for angiogenesis and vascular protection. Hydralazine also exerts reno-protective effects via its antioxidation, DNA demethylation, and anti-inflammation abilities. The above evidence provides advanced rationales for new applications of this drug beyond blood pressure lowering and arterial vasodilation. Here, we summarized the recent experimental advances in the use of hydralazine for either a vascular disease or kidney diseases, or both. Given the wide populations of people with cardiovascular and/or kidney diseases, future studies are worth validating the potential impacts of hydralazine on the clinical outcomes in selected patients.

## 1. Introduction

Hydralazine is one of the traditional antihypertensive drugs that were developed several decades ago. It is suggested as an effective arterial vasodilator that directly reduces peripheral resistance by relaxing the smooth muscle cell layer in the arterial blood vessels and has been used as an adjunct treatment for hypertension and chronic heart failure for a long time [1]. The most well-known mechanisms of hydralazine are the modulated release of purine-like compounds from sympathetic nerve endings and alterations in the Ca^2+^ balance in vascular smooth muscle [2,3]. In addition, hydralazine is suggested to prevent nitroglycerin tolerance by inhibiting the activation of membrane-bound NADH oxidase [4]. It was also indicated that hydralazine could down-regulate the NADPH oxidase expression in kidney samples from streptozotocin (STZ)-induced diabetic mice [5]. Taken together, these diverse observations suggest the complex mechanisms of hydralazine for potential cardiovascular and renal protection in addition to vasodilation that should be further addressed. As a potential rational for future clinical implications, we examined the mechanistic insights for the effects of hydralazine in individual experimental cardiovascular as well as kidney diseases in this review. We searched several keywords to find relevant references included in this review from PubMed. The keywords were inclusive of hydralazine, cardiovascular disease (such as myocardial infarction, angiogenesis, and atherosclerosis), and kidney disease (such as chronic kidney disease, acute kidney injury, and diabetic kidney disease). Studies that failed to report a specific and detailed mechanism or those not adding any novelty were excluded.

## 2. Potential Effects of Hydralazine on Cardiovascular Disease

### 2.1. Hydralazine for Cardiac Protection after Myocardial Infarction

Hydralazine is usually used as an adjunct treatment in patients with hypertension and/or chronic heart failure. While the clinical benefits of hydralazine may be related to its vasodilation and hemodynamic effects, several current studies have documented connections between hydralazine and cardiac mitochondria [6]. In a recent animal study, hydralazine was shown to reduce myocardial infarct size and protect the heart against acute ischemia/reperfusion injury (IRI) by inhibiting Drp1-mediated mitochondrial fission in isolated mouse cardiomyocytes [7,8]. It was then suggested that in addition to its antioxidant and anti-apoptotic effects, hydralazine could promote acute cardiac protection by inhibiting IRI-induced mitochondrial fission, raising the possibility of repurposing hydralazine as a novel cardioprotective therapy to improve post-infarction outcomes [7,8]. However, given the potential baroreflex effect of hydralazine, the current clinical recommendations do not support its use in ischemic heart disease. A future clinical study is critical to clarify this issue.

### 2.2. Hydralazine for Angiogenesis in Ischemic Vascular Disease

Circulating endothelial progenitor cells (EPCs) are a specific cohort of monocytes originally derived from bone marrow that may promote vascular endothelial repair and even differentiate into endothelial cells for neovascularization in various physiological and pathological processes. EPCs express CXCR4, which allows homing to neovascularization sites. Hydralazine was purposed to upregulate CXCR4 in EPCs and thereby enhance their function and capacity to home to target tissues via hypoxia-inducible factor (HIF) 1α stabilization in the presence of cardiovascular risk factors [9]. Hydralazine was also shown to stabilize HIF-1α, upregulate the vascular endothelial growth factor, and promote angiogenesis by inhibiting prolyl hydroxylases in endothelial and smooth muscle cells in vitro and in sponge angiogenesis assays in vivo [10]. Our recent study further showed that in the presence of chronic renal insufficiency, hydralazine could enhance the in vitro EPC function and improve in vivo ischemia-induced neovasculogenesis via xanthine oxidase (XO) and NADPH oxidase inhibition [11]. These preclinical observations indicate the direct pro-angiogenic effects of hydralazine on different vascular cells by multiple antioxidative mechanisms in various disease scenarios. A future clinical study may be required to validate the potential use of hydralazine for angiogenesis in ischemic vascular diseases.

### 2.3. Hydralazine for Plaque Stabilization in Atherosclerosis Disease

Regarding atherosclerosis, the antiatherogenic effect of hydralazine has been suggested in several hypercholesterolemic mice models. Hydralazine was shown to reduce the extent of atherosclerosis, which is thought to result from the in vivo mechanical effects of blood pressure reductions in apolipoprotein E-deficient mice [12]. On the other hand, hydrazine derivatives were shown to inhibit the formation of foam cells and block the carbonyl stress in smooth muscle cells in vitro [13]. Intra-plaque neovascularization is critical for the development of atherosclerotic lesions, which may lead to hemorrhage in the plaques and promote fragility and rupturing in the plaques. Hydralazine was shown to stabilize the plaques in vivo, which may be related to the inhibition of polyunsaturated fatty acid-induced oxidative stress and the oxidative stress-related angiogenic response of microvascular endothelial cells in vitro [14]. Taken together, it seems that hydralazine may retard the progression of atherosclerosis by both the direct in vitro antioxidant mechanisms and the in vivo blood-pressure-lowering effects. Given that hydralazine-induced lupus has been reported in patients with heart failure [15], future clinical studies are required to demonstrate the long-term benefits of hydralazine before it can be used for atherosclerosis cardiovascular diseases.

## 3. Potential Effects of Hydralazine on Kidney Disease

### 3.1. Hydralazine for Chronic Kidney Disease

While the majority of the effects of hydralazine are confined to cardiovascular protection, the accumulating experimental data have also revealed its substantial effects on kidney protection. It was further suggested as a potential repurposing drug, especially for chronic kidney disease (CKD) [16]. Hydralazine was shown to prevent renal damage [17] and reduce renal interstitial fibrosis as well as oxidize the low-density lipoprotein expression [18] in spontaneously hypertensive stroke-prone rats. Hydralazine could also retard the renal mesangial expansion in db/db mice with unilateral nephrectomy and an angiotensin II infusion [19]. A maternal high-fat diet may promote CKD in offspring. Hydralazine could prevent maternal obesity or dietary obesity-related CKD with attenuated renal global DNA methylation and renal fibrosis [20]. In a rat model of a metabolic syndrome, severe hypertension and proteinuria were observed in rats fed with a high-salt diet. Hydralazine was shown to alleviate hypertension, decrease urinary protein excretion, and reduce the glomerular sclerosis level [21,22]. Accordingly, it seems that hydralazine may provide effective renal protection across different CKD models. Given the potential contribution and universal presence of hypertension during the progression of CKD in both animal models and clinical settings, it is possible that the renal protection of hydralazine is mainly related to its blood-pressure-lowering effects. Further experiments may be required to elucidate the potential role of hydralazine in the direct reno-protective mechanisms for CKD.

### 3.2. Hydralazine for Acute Kidney Injury

Hydralazine was shown to demethylate RASAL1 via reno-protective effects in a mouse model of acute kidney injury (AKI) with IRI. Meanwhile, hydralazine-induced CpG promoter demethylation followed attenuated renal fibrosis and preserved the renal function independently of its blood-pressure-lowering effects in a mouse model of AKI-to-CKD progression [23,24]. Additionally, hydralazine attenuated renal inflammation in diabetic rats with AKI by decreasing the advanced glycation end product (AGE)-induced receptors for AGEs (RAGEs), inducible nitric oxide synthases (iNOSs), and COX-2 expression in rat mesangial cells in vitro, and by reducing the inflammation-related proteins as well as the JAK2/STAT3 signaling pathways in rat kidney tissues in vivo [25]. Furthermore, hydralazine reduced renal IRI through inhibiting nitric oxide (NO) production via the iNOS/NO pathway; reducing oxidative stress and inflammatory proteins such as tumor necrosis factor (TNF) α, interleukin (IL) 1β, and IL-6; and decreasing apoptosis via a mitochondrial-dependent pathway [26]. Accordingly, it seems that systemic antioxidation and/or anti-inflammation mechanisms, rather than blood pressure lowering, may contribute to the direct reno-protective effects of hydralazine in AKI, which may be different from that in CKD.

### 3.3. Hydralazine for Diabetic Kidney Disease

Diabetic kidney disease (DKD) is one of the major causes of end-stage renal disease. Different from other chronic renal diseases, DKD is characterized by the early presence of glomerular hyper-infiltration and hyper-albuminuria, which may be associated with significant systemic inflammation and are largely influenced by fluctuations in blood sugar and blood pressure. Hydralazine decreased protein glycation and down-regulated RAGE, NADPH oxidase, and super oxide dismutase expression in the kidneys of STZ-induced diabetic mice [5]. A hydralazine treatment also partially attenuated the albuminuria in type 2 diabetic Otsuka Long–Evans Tokushima fatty rats [27]. In a rat model of obesity-related kidney disease, mesangial expansion was reduced with hydralazine treatments [28]. In another DKD animal model with a strain of spontaneously hypertensive/NIH-corpulent rats, the renal pentosidine content was correlated with the development of proteinuria. Hydralazine not only reduced the renal pentosidine content and attenuated glomerular damage in vivo, but also inhibited the formation of AGE in vitro [29]. Furthermore, we recently showed that hydralazine could protect renal proximal tubular epithelial cells against the insults of high glucose in vitro and prevent the progression of DKD in vivo via antioxidation and anti-inflammation mechanisms, including XO and NADPH oxidase inhibition and nuclear factor erythroid 2-related factor 2/heme oxygenase 1 activation [30]. Taken together, hydralazine may exert reno-protective effects via multiple antioxidation and anti-inflammation mechanisms in various animal models of DKD. Given the absence of hypertension and minimal changes to blood pressure, the beneficial actions of hydralazine on renal function may be independent of blood pressure in these DKD animal models.

## 4. Potential Mechanisms for Beneficial Effects of Hydralazine

### 4.1. Potential Mechanisms for Systemic Effects of Hydralazine

**Figure 1:**
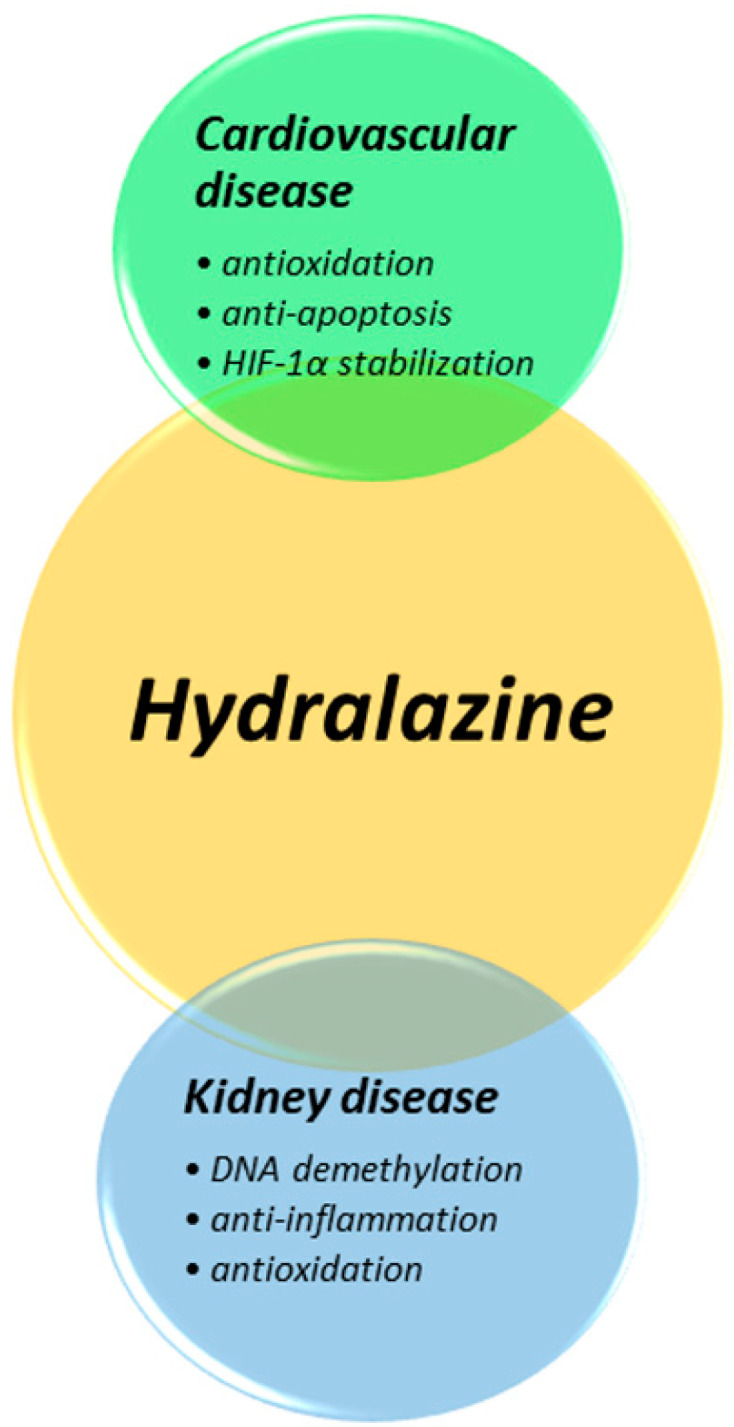
Overview of the systemic impacts of hydralazine and its potential mechanisms.

Recent experimental data suggest some novel cardiovascular and renal protective effects of hydralazine, which may be related to multiple and differential mechanisms in each individual disease (Figure 1). The accumulating evidence suggests that hydralazine may exert cardiovascular protection rather than its blood-pressure-lowering effects. On the other hand, in addition to its blood-pressure-lowering effect, some tissue-specific mechanisms such as antioxidation, anti-inflammation, and DNA demethylation may contribute to the reno-protective effects of hydralazine. While the presence of hypertension may vary and blood pressure changes in different animal models are not consistent, the cardinal mechanism for the universal effects of hydralazine on cardiovascular and renal protection may be related to its direct tissue-protection effects, such as its antioxidative ability.

### 4.2. Potential Mechanisms for Intracellular Effects of Hydralazine

**Figure 2:**
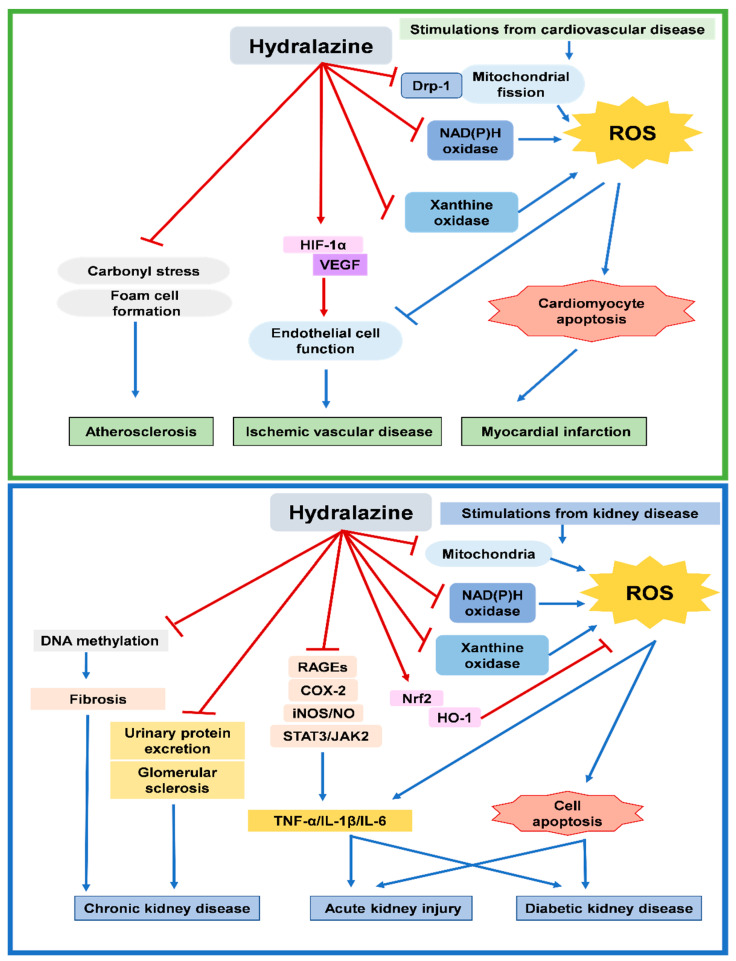
Overview of mechanisms of hydralazine for antioxidation in cardiovascular and kidney diseases. Heme oxygenase 1: HO-1; hypoxia inducible factor 1α: HIF-1α; inducible nitric oxide synthase: iNOS; interleukin: IL; vascular endothelial growth factor: VEGF; nitric oxide: NO; nuclear factor erythroid 2-related factor 2: Nrf2; reactive oxygen species: ROS; receptor for advanced glycation end products: RAGE; tumor necrosis factor α: TNF-α.

The intracellular mechanisms for the antioxidative capacity of hydralazine may be complex, which could be related to its functions of mitochondrial inhibition, NADPH oxidase inhibition, XO inhibition, and nuclear factor erythroid 2-related factor 2/heme oxygenase 1 activation. Hydralazine was also shown to have anti-apoptosis and HIF-1α stabilization capabilities. Furthermore, hydralazine was shown to down-regulate RAGE, iNOS, COX-2, TNF-α, IL-1β, and IL-6 signaling via both redox-dependent and -independent mechanisms (Figure 2). Future studies may be required to clarify the potential interaction of these complex intracellular mechanisms.

## 5. Conclusions

While hydralazine has been used for a long time in the treatment of hypertension and chronic heart failure, its clinical mechanisms remain to be further explored. Given the limited evidence directly supporting its mechanisms in each specific disease, here we attempted to summarize the general beneficial effects, especially in cardiovascular and renal diseases. Altogether, the experimental evidence suggests novel and complex antioxidative and anti-inflammatory mechanisms of hydralazine, including mitochondrial inhibition, NADPH oxidase inhibition, XO inhibition, heme oxygenase induction, cytokine modulation, and others for both cardiovascular and renal protection. The potential impacts of hydralazine other than vasodilation may provide this old drug with a new scenario.

These experimental studies open up the possibility of repurposing hydralazine as a potential cardio- and reno-protective agent through its antioxidative and anti-inflammatory mechanisms. Further investigations may be indicated to verify whether the potential clinical benefits of hydralazine could be related to its antioxidative and anti-inflammatory capacity, particularly in patients with high levels of ROS and/or inflammation. In this regard, the proper doses and regimens should be also established. Given the worldwide prevalence and clinical significance of cardiovascular as well as kidney diseases, future studies are worthwhile to validate the potential impacts and long-term safety of hydralazine as a complex antioxidant in selected clinical diseases.

## Abbreviations

AGE	Advanced glycation end product
AKI	Acute kidney injury
CKD	Chronic kidney disease
DKD	Diabetic kidney disease
EPC	Endothelial progenitor cells
HIF	Hypoxia-inducible factor
iNOS	Inducible nitric oxide synthase
IL	Interleukin
IRI	Ischemia/reperfusion injury
NO	Nitric oxide
RAGE	Receptor for advanced glycation end products
STZ	Streptozotocin
TNF	Tumor necrosis factor
XO	Xanthine oxidase
